# Machine learning assisted optimization of electrochemical properties for Ni-rich cathode materials

**DOI:** 10.1038/s41598-018-34201-4

**Published:** 2018-10-25

**Authors:** Kyoungmin Min, Byungjin Choi, Kwangjin Park, Eunseog Cho

**Affiliations:** 1 Platform Technology Lab, Samsung Advanced Institute of Technology, 130 Samsung-ro, Suwon Gyeonggi-do, 16678 Republic of Korea; 2 Energy Lab, Samsung Advanced Institute of Technology, 130 Samsung-ro, Suwon Gyeonggi-do, 16678 Republic of Korea; 3 Department of Mechanical Engineering, Gachon University, 1342 Seongnamdaero, Gyeonggi-do, 13120 Republic of Korea

## Abstract

Optimizing synthesis parameters is the key to successfully design ideal Ni-rich cathode materials that satisfy principal electrochemical specifications. We herein implement machine learning algorithms using 330 experimental datasets, obtained from a controlled environment for reliability, to construct a predictive model. First, correlation values showed that the calcination temperature and the size of the particles are determining factors for achieving a long cycle life. Then, we compared the accuracy of seven different machine learning algorithms for predicting the initial capacity, capacity retention rate, and amount of residual Li. Remarkable predictive capability was obtained with the average value of coefficient of determinant, R^2^ = 0.833, from the extremely randomized tree with adaptive boosting algorithm. Furthermore, we propose a reverse engineering framework to search for experimental parameters that satisfy the target electrochemical specification. The proposed results were validated by experiments. The current results demonstrate that machine learning has great potential to accelerate the optimization process for the commercialization of cathode materials.

## Introduction

Machine learning (ML) algorithms have been suggested as the most innovative methodology in recent years because of their great potential. One of reasons is that since a tremendous amount of datasets have been generated and accumulated, researchers now have the opportunity to utilize these databases, which can be trained with ML algorithms to model unexplored areas of parameter space. Various applications have been successfully demonstrated in this field such as autonomous driving cars^1^, face recognition^2^, and image segmentation/detection^3^. Further, the promising future of ML techniques is vastly expanding to the area of materials science. For example, ML methods have already proven capable of predicting band gaps, dielectric constants, and the stability of semiconductors, dielectric materials, polymers, and molecules^4–9^ These previous results have demonstrated that applying ML is particularly beneficial for finding new materials with improved properties. In addition, ML can be applied to develop interatomic potentials for running molecular dynamics simulations^10,11^, and to predict possible chemical reactions^12^ or thermodynamic stability^13^.

Despite these successes, the applications of ML are still limited because providing a reliable database is a prerequisite before any ML algorithm can be implemented because the accuracy of the trained model is solely dependent on the quality and amount of training data. In this respect, many studies have focused on building datasets from calculated results with high-throughput simulations^14,15^. However, the transferability of constructed models based on simulation data is limited because the correlation with the actual device performance is often unclear. In this regard, it would be preferable if more datasets from experiments were industrially available; then, ML methods could be expected to provide much more reliable output.

In this respect, we herein apply ML algorithms to an experimental database to improve the electrochemical performance of Ni-rich cathode materials, LiNi_x_Co_1-x-y_Mn_1-x-y-z_O_2_ (NCM) with x> 0.85, by optimizing their synthesis parameters. Ni-rich NCM has been considered as a promising cathode material for electric vehicle applications because of its large capacity and cheaper manufacturing cost^16^. In this regard, designing an NCM material with a larger capacity, longer cycle life, and lower amount of residual Li is a key strategy for its commercialization, which will allow the development of the next generation of electric vehicles. This poses several technical challenges, including a number of synthesis parameters that need to be tuned, such as calcination temperature, particle size distribution, washing process, dopant concentrations, and coating materials. Each process can have a significant impact on the electrochemical properties of the final cathode. For example, the washing process can lessen the amount of unwanted residual Li in the cathode. In addition, by including electrochemically stable doping elements, the structural stability can be greatly enhanced^16,17^. With the coating process, direct contact between the cathode surface and the electrolyte could be prevented, leading to improved electrochemical performance^16,18,19^. This is made more challenging by the fact that even small modifications of each parameter could result in significant variations in electrochemical performance. Thus, optimizing all variables simultaneously is difficult, and can only be achieved with experience and knowledge. However, with the aid of ML algorithms, it is now feasible to search all possible experimental datasets by providing parameters to a trained prediction model, which greatly accelerates the optimization process.

In this study, we first perform basic statistical analysis to distinguish which experimental parameters are most correlated with electrochemical performance. Then, an ideal ML model for predicting electrochemical properties is constructed by comparing its accuracy with various types of regression algorithms. After validating the trained model, it is further expanded to implement a reverse engineering framework to suggest the ideal experimental parameters, which can satisfy the target electrochemical specifications and the results are validated with experiments.

## Methods

### Machine learning model

In order to choose an ML algorithm with the best performance, seven different types of ML regression models are employed: support vector machine (SVM), decision tree (DT), ridge regression (RR), random forest (RF), extremely randomized tree (ERT), and neural network (NN) with multi-layer perceptron. The adaptive boosting (AdaBoost) algorithm was further embedded into the ERT model. The python-based ML package Scikit-learn^20^ was used to implement these models. Details of the theoretical background for each algorithm are not discussed here because they can be easily found elsewhere^20^. To improve the prediction accuracy, the randomized search algorithm was implemented to find the optimal hyperparameters for each model. The NN model consisted of five layers with 10 nodes each. The ML model was cross-validated with a randomly chosen 80% of the dataset (train set) to establish the prediction model, and the remaining 20% (test set) was used to validate the constructed model.

### Database

**Figure 1:**
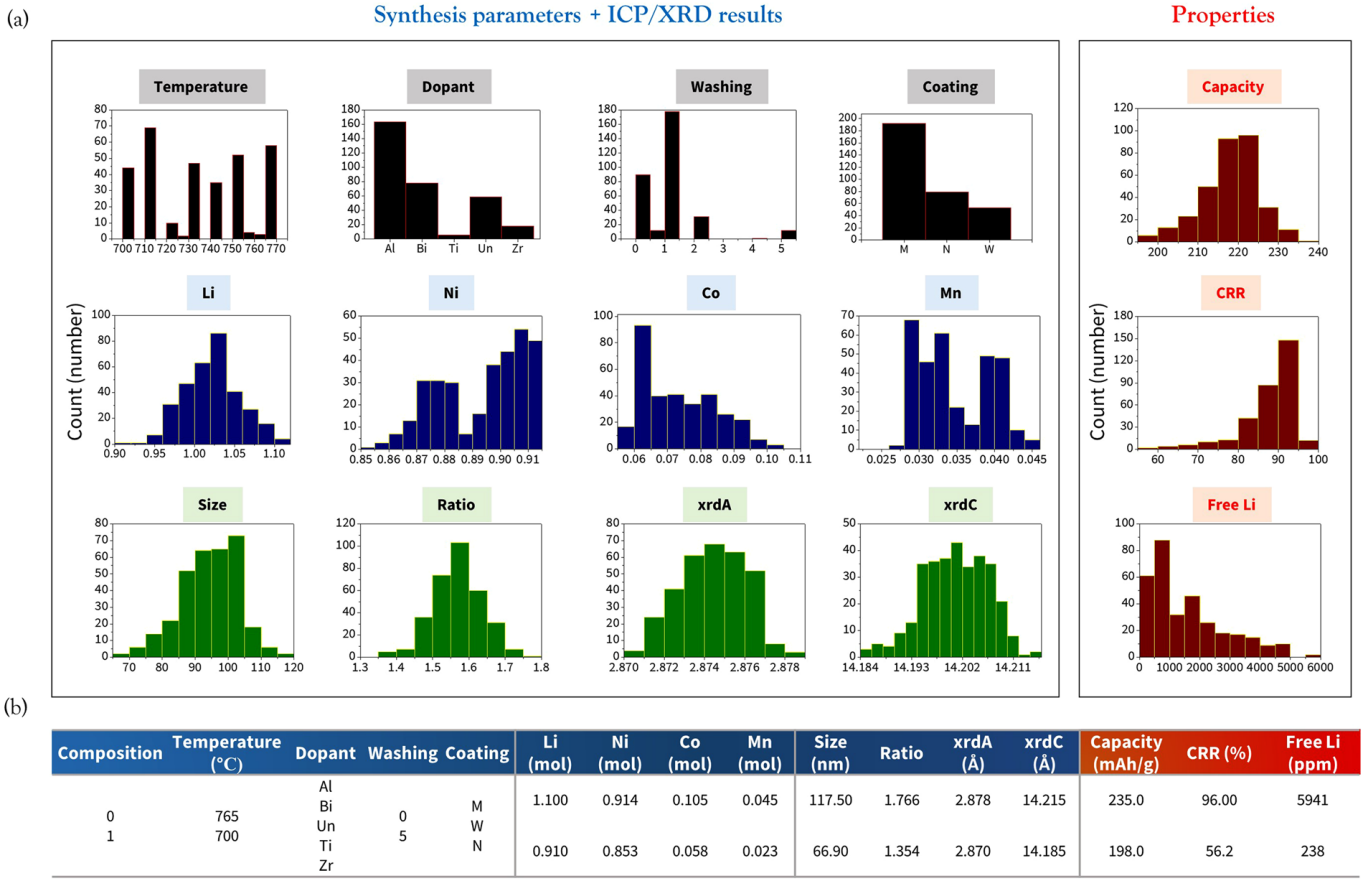
(**a**) Distribution and (**b**) the range of experimental synthesis parameters, ICP data, XRD results, and electrochemical properties.

A total number of 330 experimental datasets for Ni-rich NCM cathodes whose Ni content is more than 85% were constructed with 13 input variables (synthesis parameters + inductively coupled plasma mass spectrometry (ICP-MS) + X-ray diffraction (XRD) results) and three output variables (initial capacity, cycle life, and the amount of residual Li). The general synthesis process and electrochemical testing methods can be found in our previous work^18,19^. The distributions of these parameters over the whole dataset are shown in Fig. 1(a,b). First, among the various synthesis parameters, five principal variables (composition, calcination temperature, dopant, washing, and coating materials) were selected when constructing the ML model. This is because the values of the other variables such as the machine parameters during the drying process, washing time, and second calcination temperature after the coating process are almost the same throughout the dataset; thus, those factors do not significantly affect the accuracy of the models.

A brief description of each experimental variable is shown in Table 1. The composition indicates whether the size distribution of the agglomerated secondary particles of the NCM cathode is unimodal (0) or bimodal (1). Temperature is the first calcination temperature after providing the precursors for synthesis. The dopant variable can be aluminum (Al), undoped (Un), titanium (Ti), zirconium (Zr), or doping more than two materials (Bi). The washing variable indicates the mass ratio of water to active materials of NCM; a value of 0 means that the washing process is not conducted. For coating materials, each character indicates materials (M), water evaporation (W), or none (N). The major types of applied coating materials among the 330 experimental datasets are Co_3_(PO_4_)_2_, Mg_3_(PO_4_)_2_, and their mixture as (CoMg)_3_(PO_4_)_2_. When constructing the ML inputs, these coating materials are denoted only as ‘M’ for simplicity. This is because several parameters need to be provided to distinguish the performance of each coating material, including the total amount, the ratio of Co- to Mg-phosphate, coating temperature, and the time, which would require a much more data. Moreover, other types of coating materials such as Co-, Al-, and Zr-oxides are also included in some datasets, which makes it even more difficult to construct our database. In general, the coating process for current experimental datasets is as follows. (1) The metal and phosphate source is provided and dissolved in deionized water. (2) NCM powder is added in the solution and dried. (3) Finally, they are heated at around 700~800 °C for 0.5 to 5 hours. More details can be found in previous references^18,19^.

**Table 1:** Input and output variables for construction of the ML model with short descriptions.

Variables	Description	
Input	Composition	0: Unimodal, 1: Bimodal
	Temperature	The first calcination temperature
	Dopant	Al: Aluminum, Un: Undoped, Ti: Titanium, Zr: Zirconium Bi: doping more than two materials
	Washing	Mass ratio of water to the active materials
	Coating	M: Materials (Co_3_(PO_4_)_2_, Mg_3_(PO_4_)_2_,..), W: Water evaporation, N: None
	ICP	The amount of Li, Ni, Co, and Mn
	XRD	Size: the primary particle size, Ratio: ratio of (003) to (104) peaks xrdA: lattice parameter a, xrdC: lattice parameter c
Output	Capacity	The first discharge capacity at 0.2 C
	CRR	Capacity retention rate after 50 cycles at 1 C
	Free Li	The amount of residual Li after initial synthesis

The ICP results show the amounts of atomic elements in the NCM materials. From the XRD analysis, the size of the primary particles in the NCM (size), the peak ratio of the (003) to (104) reflections in the XRD pattern (ratio), lattice parameter *a* (xrdA), and lattice parameter *c* (xrdC) are collected. Finally, for the electrochemical properties, the initial discharge capacity (capacity) at a C-rate (the rate of discharging the cathode from its maximum capacity) of 0.2 C, the cycling retention rate at 1 C after 50 cycles (CRR), and the amount of residual Li compounds (free Li) after the initial synthesis were obtained. We note that controlling the amount of residual Li is critical because excessive Li could lead to slurry gelation, which results in a non-uniform surface shape during slurry deposition on the current collector^21,22^.

## Results and Discussions

### Correlation between variables

**Figure 2:**
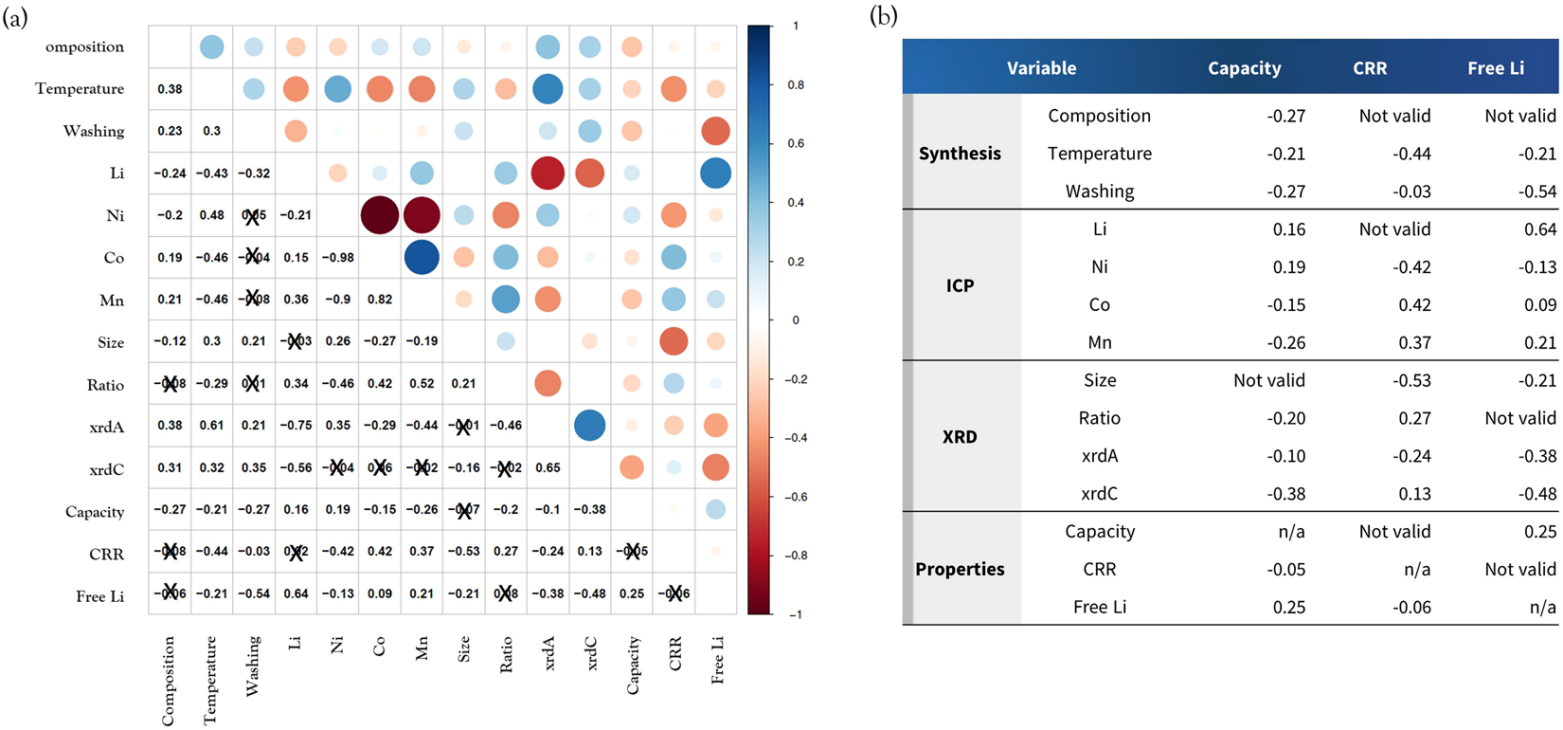
(**a**) Pearson correlation coefficient (R) between all variables and (**b**) electrochemical properties. Values marked with an X indicates that this p-value is not valid (>0.05).

To provide a general statistical overview of the dataset, the Pearson correlation coefficient (R) was calculated between variables except for text variables (dopant and coating materials), as shown in Fig. 2(a). The color and the size of the circles in the diagram represent the magnitude and the direction of the correlation. It is important to note that hardly any strongly correlated values (R > 0.7) were found, indicating that the relationships between variables cannot be explained with a simple linear function. Hence, the current results should be used to obtain general trends in the experimental parameters. The probability values (P-values) were also calculated and the data points with P-values larger than 0.05 are marked with an ×, indicating a lack of statistical confidence.

From the R values, one can see how the variables are linearly correlated; they can be between +1 and −1 where a positive (negative) number indicates that variable A increases as variable B increases (decreases). It is important to note that in-depth investigation is necessary to determine meaningful relations between some parameters. For example, the relation between the synthesis parameters and the ICP results is not critical because these quantities are provided as inputs. In this regard, one should not be misled by the strong correlation (R = −0.98) between Ni and Co as an example. (The amount of Co must be reduced to increase the Ni content to satisfy the stoichiometry of NCM.) Similarly, the moderate correlation (R = −0.46) between temperature and Co does not necessarily mean that increasing calcination temperature decreases the amount of Co. In addition, modification of the synthesis parameters can considerably affect the XRD results as follows. (1) Providing more Li could decrease the lattice parameters (R = −0.75 and −0.56 for xrdA and xrdC, respectively). (2) Increasing the synthesis temperature generally increases the lattice parameters. (R = 0.61 and 0.31 for xrdA and xrdC, respectively).

It is more critical to investigate the correlations between experimental variables and the electrochemical properties, as shown in Fig. 2(b). First of all, none of the variables are strongly correlated (R > 0.7) with the target properties, indicating that this dataset cannot be simply explained with a linear relation. Hence, the R values here should be used to obtain general trends in the dataset. Especially for the initial capacity, all of the R values are too small (R < 0.4) to suggest a correlation. For the CRR results, structures with a higher calcination temperature, higher Ni content, and a larger size of the primary particle resulted in poorer performance. This can be explained from the peak ratio of the (003) and (104) planes (*i*.*e*., the crystallinity of the NCM structure), which decreases with higher Ni content (R = −0.46) but increases with higher Mn (R = 0.52) and Co content (R = 0.42). It is well-known that Ni-rich NCM with better crystallinity (larger peak ratio, less-disordered) can effectively mitigate degradation behaviors^16^. This is because a low peak ratio originates from a more disordered material due to transition metal occupation of the lithium layer, which can hinder Li diffusion and also lead to a phase transformation from the layered oxide to the spinel phase. For the free Li, its relation is already predictable because residual Li will be removed by the washing process (R = −0.54) and the ICP results for Li are directly correlated with this value (R = 0.64).

### Construction of prediction model using machine learning algorithm

**Figure 3:**
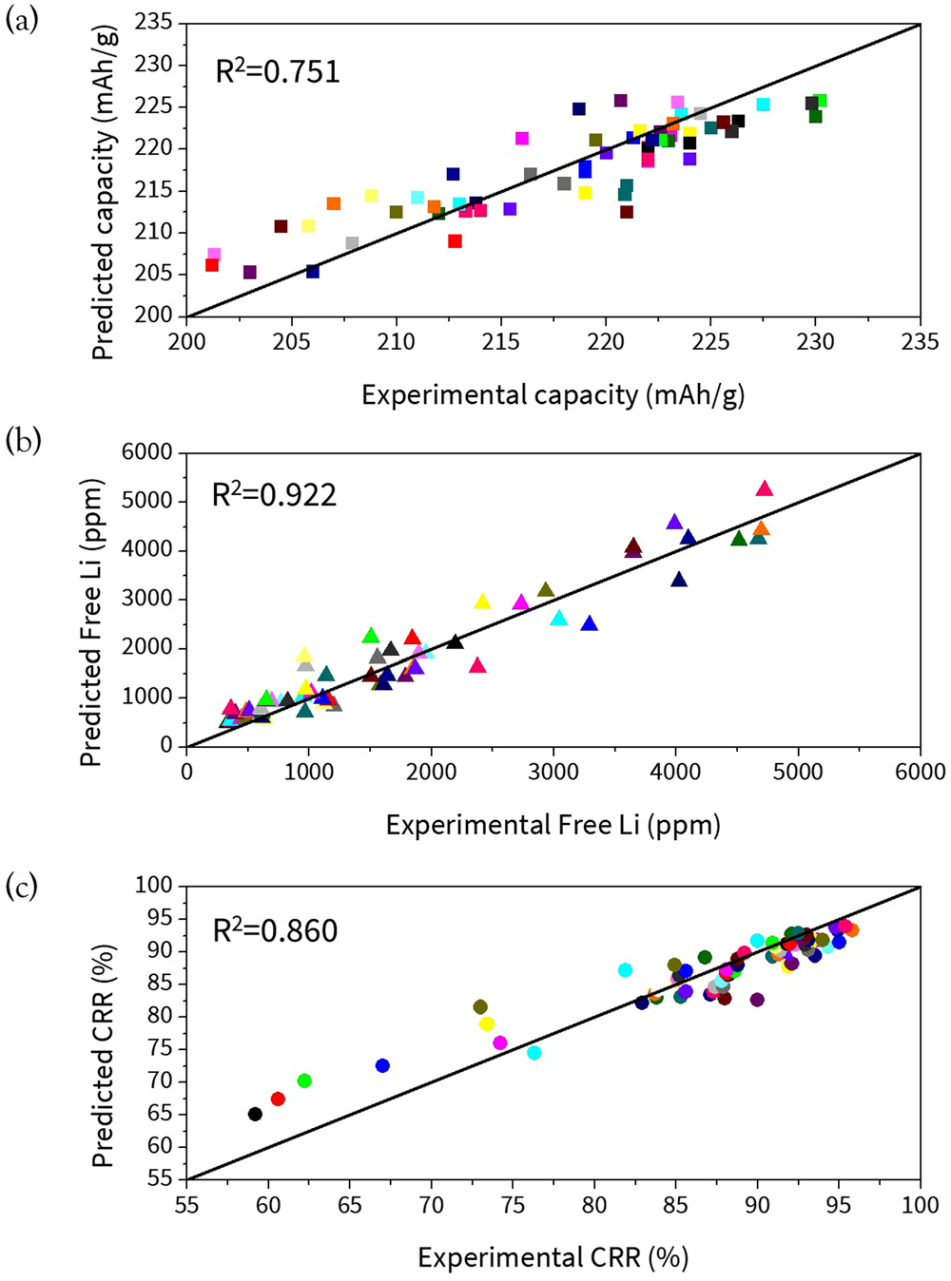
Experimental properties vs. predicted properties (test set) for initial capacity, free Li, and CRR from ERT with AdaBoost model with the maximum R^2^ value.

To construct a prediction model for electrochemical properties based on the experimental parameters, it is important to choose an optimal ML model from among the various types of ML regression algorithms, which can represent the current dataset with the best accuracy. First, to obtain statistically meaningful results, we randomly selected 300 different training sets because there are more than thousands of sets available to choose from the 80% of the 330 training datasets. After training each ML model with these training sets, the constructed model was validated with the remaining test set (20%) by calculating the average, maximum, and standard deviation (STD) of the coefficient of determinant (R^2^) value, as shown in Table 2. In terms of the performance of each model, we first note that the model with a larger average value of R^2^ usually showed a larger maximum value and a smaller STD value. More importantly, the ensemble methods (RF and ERT) exhibited superior accuracy compared to the linear model, NN, and others. To conclude, among several regression models, the ERT + AdaBoost algorithm was found to predict the electrochemical properties with the best accuracy, whose maximum R^2^ value and mean absolute error (in the parenthesis) were 0.751 (2.84 mAh/g), 0.922 (289.18 ppm), and 0.860 (2.33%) for the initial capacity, free Li, and CRR, respectively. The comparison between experimental results vs. predicted properties from the test set based on this model is shown in Fig. 3.

**Table 2:** Average, maximum, and standard deviation (STD) of R^2^ values from each regression model using 300 randomly chosen datasets for the initial capacity, capacity retention rate (CRR), and the amount of free Li.

Regression model	Capacity			CRR			Free Li		
	Average	Max	STD	Average	Max	STD	Average	Max	STD
Decision Tree	0.290	0.602	0.108	0.412	0.716	0.139	0.690	0.894	0.161
Ridge Regression	0.286	0.479	0.090	0.507	0.685	0.104	0.722	0.842	0.078
Support Vector Regression	0.518	0.737	0.109	0.645	0.838	0.119	0.721	0.856	0.072
Random Forest	0.443	0.654	0.108	0.601	0.831	0.145	0.705	0.941	0.211
Neural Network (10, 5)	0.391	0.700	0.139	0.592	0.839	0.160	0.787	0.902	0.062
Extremely Randomized Tree	0.540	0.739	0.094	0.666	0.846	0.114	0.820	0.914	0.058
Extremely Randomized Tree + AdaBoost	0.576	0.751	0.064	0.707	0.860	0.063	0.842	0.922	0.034

An important perspective can be gained from the above results. Overall, it is noted that the prediction capability is the best (R^2^ ~ 0.94) for the free Li. This could be because we already include information related to the amount of Li from the ICP measurement as an input variable, which is directly associated to the free Li. On the other hand, the predictive accuracy for the initial capacity was shown to be the worst (R^2^ < 0.75) but we note that implementing a ML method is still advantageous because its prediction capability for CRR is great (R^2^ ~ 0.86). This is important because in general, a larger initial capacity can be achieved by increasing the Ni content but a Ni-rich NCM cathode always suffers from capacity loss during electrochemical cycling due to various degradation behaviors^16,23^. This fact greatly limits the commercialization of Ni-rich NCM for electric vehicles. Furthermore, since measuring the CRR is the most time-consuming process, as approximately four days (100 hours) are required to obtain the CRR value at the 50^th^ cycle under 1 C, the cost of optimizing the experimental parameters to synthesize NCM with a greater cycle life can be largely saved by employing this prediction model.

### Reverse engineering to satisfy target specifications

**Figure 4:**
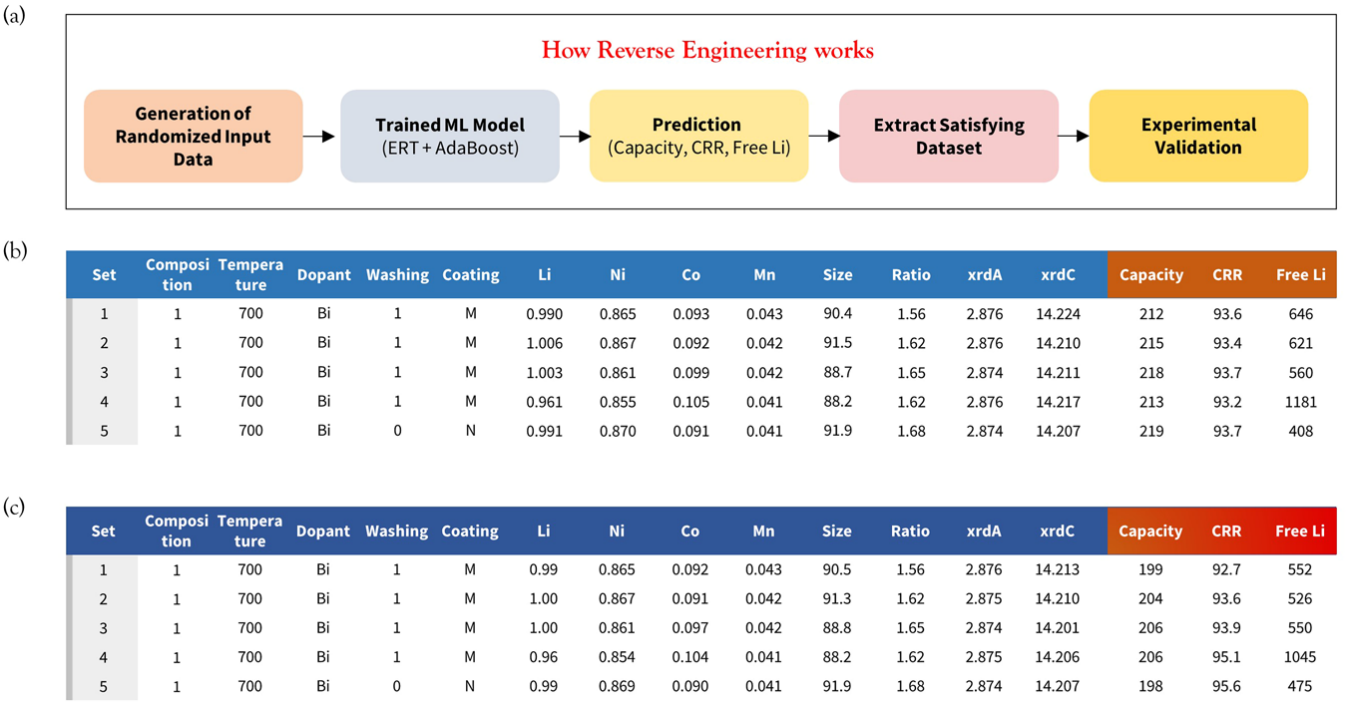
(**a**) Flowchart depicting the reverse engineering scheme. (**b**) Proposed synthesis parameters, ICP, and XRD results to satisfy the proposed target specifications (CRR > 93% and Free Li < 1300 ppm) among 50,000 datasets and (**c**) their experimental validation.

The establishment of an accurate prediction model enables researchers to perform experiments synthesizing NCM materials in a computer-aided artificial lab with various combinations of experimental parameters and thus further improve their performance in a reduced amount of time. More importantly, the ultimate goal of constructing this prediction model should be to propose optimized experimental parameters that satisfy the target specifications. We call this process reverse engineering and the flowchart is shown in Fig. 4(a). First, we generate the input data from 50,000 datasets by randomly choosing the parameters within the range for each variable shown in Fig. 1(b). We note that since the trained ML algorithm used for extrapolation is not strongly validated, we only search for optimal parameters within the range of the trained database (interpolation) shown in Fig. 1(b). Then, these are provided to the trained ML model (ERT + AdaBoost) and the corresponding electrochemical properties are predicted. Since the predictive capability is lowest for the initial capacity (R^2^ = 0.751), we prioritize the other two categories and extract the datasets satisfying the target specifications (CRR > 93%, free Li < 1300 ppm). We claim that these criteria are the minimum necessary for commercial applications. For example, a free Li concentration greater than 1300 ppm could result in easy gelation in the cathode slurry during the manufacturing process based on our experience. Finally, experimental validation for the suggested parameters is achieved.

The satisfying experimental datasets are shown in Table SI, Supporting Information (SI). Some interesting perspectives can be addressed based on these results. (1) The calcination temperature for most of the sets is around 700~710 °C. (2) Using a Zr dopant or using more than two dopant materials seems to be effective. (3) A washing process is always preferred. (4) The evaporation method (washing then calcination again) is effective. (5) A Ni content of more than 0.87 is not preferable. We note that although the reverse engineering process provides a set of optimized parameters within the constructed ML prediction model, different constraints could be applied depending on how researchers want to design the synthesis process. For example, although a lower temperature is preferable within our model, previous works still applied calcination temperatures of around 750 °C for Ni-rich NCM^18,19^. However, in those cases, the grain boundaries should be clearly constructed and the washing process needs to be omitted to simplify the synthesis process. Therefore, one might want to add more datasets (no washing, coating only) that can properly describe this process with more details.

For the proposed data sets, experimental validation was performed. It is important to note that conducting experiments with given synthesis parameters and elemental concentrations is straight-forward but matching XRD results is not an easy task because they are strongly correlated with the provided inputs. In this regard, we were able to synthesize only five cases that were close to the suggested data, among 15 proposed sets. (Fig. 4(b,c)) The experimental results for the first charge-discharge curve at 0.1 C and the CRR change during cycling are shown in Fig. S.1, SI. Those five cases exhibited great performance (*e*.*g*., set #4 showed a capacity of 206 mAh/g with a CRR of 95.1% and free Li of 1045 ppm). Al and Zr were used as dopant materials (Bi) and (CoMg)_3_(PO_4_)_2_ was provided as a coating material. The average difference between the proposed and actually synthesized materials in terms of input parameters (ICP and XRD) was only 0.2%. For the electrochemical properties, the average differences were 6.3%, 1.0%, and 12.8% for the capacity, CRR, and free Li, respectively. This validation indicates that the current model can potentially guide the optimization of the cathode materials synthesis process and is effective for finding the ideal parameters.

It is important to address potential avenues for improving the current method. As discussed, the present prediction model has intrinsic limitations regarding its input parameters. First, for the XRD data, a possible solution for this problem could be constructing an ML prediction model without the XRD dataset. If this works, it would also be advantageous because the measurement time required for XRD analysis could be omitted. In this respect, ERT with AdaBoost algorithm was further applied to construct a prediction model without using the XRD data. Unfortunately, such an approach reduced the R^2^ values significantly from their original accuracies of 0.751 to 0.606, 0.922 to 0.881, and 0.860 to 0.702 for the capacity, free Li, and CRR, respectively. Although those reduced R^2^ values are still moderately accurate, further improvement is necessary to apply this model to commercial processes to lessen the possibility of misleading experiments. Second, more detailed input parameters should be provided, although this will require many more datasets, *i*.*e*., actual combinatorial data sets for bi-doping cases, coating materials, as well as duration and temperature ranges for the coating process, washing process, and so on. This will make it possible to control the synthesis process in more sophisticated way.

To summarize, we believe the current approach can be used to accelerate the optimization of synthesis parameters as follows. (1) The accumulation of datasets always occurs during the design of experiments. (2) Based on these datasets, one can construct a prediction model based on ML. (3) If the ML model can predict with acceptable accuracy, all of combinations of input variables can be easily predicted. (4) These results can help researchers to understand which variables are the most critical and how they can be modified. (5) The model can be fine-tuned by performing experiments based on the proposed sets of parameters.

## Conclusions

In this study, we have demonstrated that ML algorithms can be implemented for predicting the electrochemical properties of Ni-rich NCM cathode materials based on an experimental database. The database was compiled from 330 experimental datasets, which were obtained in a controlled and consistent environment. First, the correlation values indicate that structures with higher calcination temperatures, higher Ni content, and a larger primary particle size result in poorer performance in terms of cycle life. Among the seven different ML regression models that were tested, the ERT with AdaBoost algorithm exhibited the best performance (largest R^2^ score) for predicting the initial capacity, residual Li, and the cycle life. Finally, a reverse engineering scheme was conducted to propose ideal experimental parameters to fulfill the target specifications. Then, the proposed sets were further validated with experiments. The current study demonstrates that ML algorithms can successfully contribute to the search for an ideal synthesis process of Ni-rich cathode materials, leading to accelerated development of lithium ion batteries with higher capacity and longer cycle life for electric vehicles.

## Electronic supplementary material

Supplementary Information

## Data Availability

The data will be available upon reasonable request to the corresponding authors.
