# Supplementary material for: Machine learning assisted optimization of electrochemical properties for Ni-rich cathode materials
Source: Sci Rep. 2018 Oct 25;8:15778. doi: 10.1038/s41598-018-34201-4 (PMC6202356; doi:10.1038/s41598-018-34201-4)
Supplement: Supplementary file 1 — Supplementary Information [file 41598_2018_34201_MOESM1_ESM.docx]

Supporting Information

Machine learning assisted optimization of electrochemical properties for Ni-rich cathode materials

Kyoungmin Min^1,†,*,^, Byungjin Choi^2,†^_,_ Kwangjin Park^3^_,_ and Eunseog Cho^1,*^

^1^Platform Technology Lab, ^2^Energy Lab, Samsung Advanced Institute of Technology, 130 Samsung-ro, Suwon, Gyeonggi-do, 16678, Republic of Korea.

^3^Department of Mechanical Engineering, Gachon University, 1342 Seongnamdaero, Gyeonggi-do, 13120, Republic of Korea

**
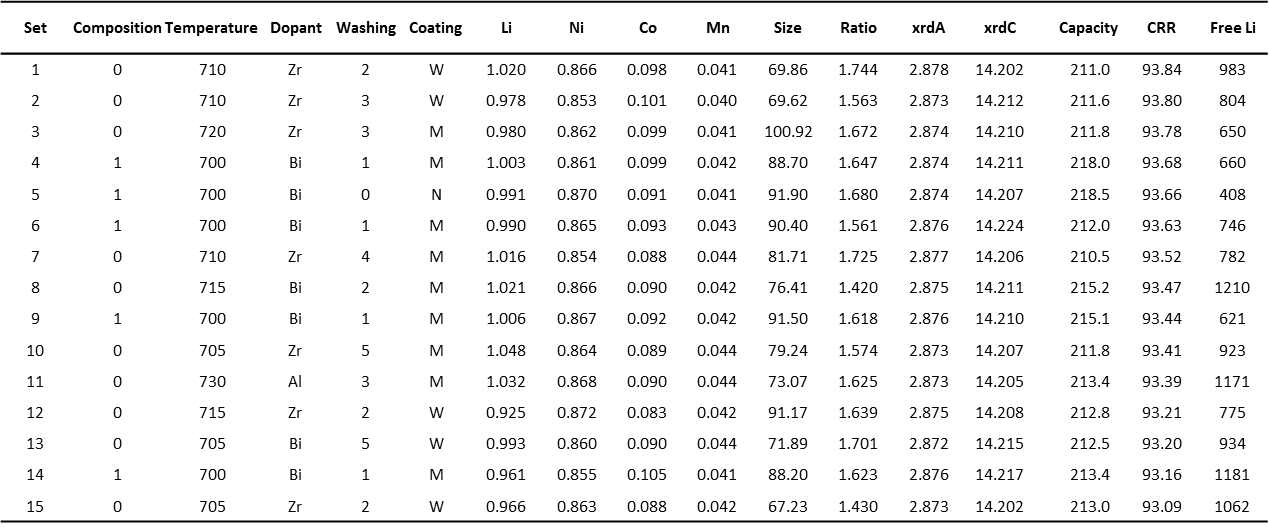
**

Table S.I. Top 15 of the proposed synthesis parameters, ICP, and XRD results for satisfying the proposed target specifications (CRR > 93% and Free Li < 1300 ppm) among 50,000 datasets.

**
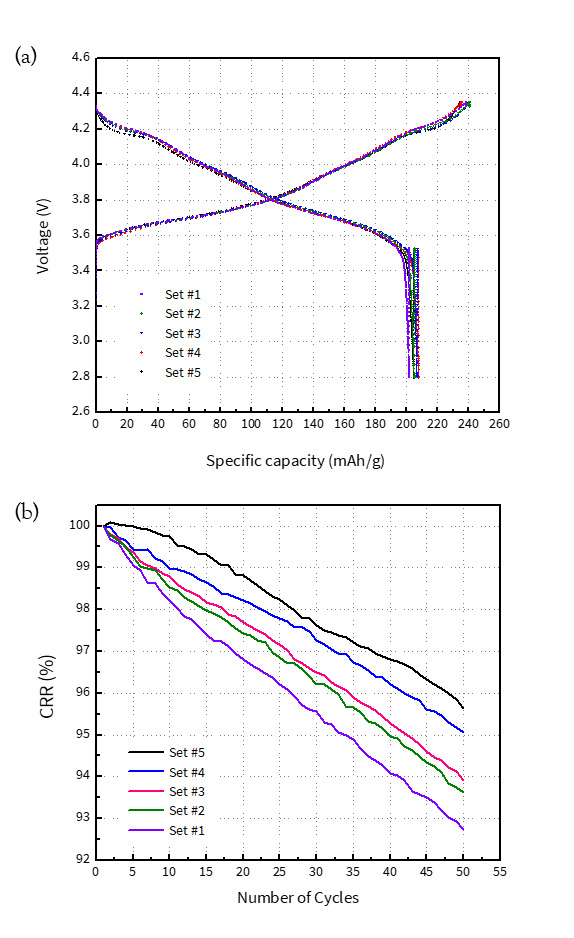
**

Figure S.1. (a) The first charge and discharge curve at 0.1 C and (b) the capacity retention rate for the experimental sets in Figure 4(c).
